# Recruitment of LC3 by *Campylobacter jejuni* to Bacterial Invasion Site on Host Cells *via* the Rac1-Mediated Signaling Pathway

**DOI:** 10.3389/fcimb.2022.829682

**Published:** 2022-03-03

**Authors:** Shiho Fukushima, Takaaki Shimohata, Yuri Inoue, Junko Kido, Takashi Uebanso, Kazuaki Mawatari, Akira Takahashi

**Affiliations:** ^1^Department of Preventive Environment and Nutrition Institute of Biomedical Sciences, Tokushima University Graduate School, Tokushima, Japan; ^2^Faculty of Marine Biosciences, Fukui Prefectural University, Fukui, Japan

**Keywords:** *campylobacter jejuni*, autophagy, LC3, Rac1, invasion

## Abstract

*Campylobacter jejuni* is a leading cause of food-borne disease worldwide. The pathogenicity of *C. jejuni* is closely associated with the internalization process in host epithelial cells, which is related to a host immune response. Autophagy indicates a key role in the innate immune system of the host to exclude invasive pathogens. Most bacteria are captured by autophagosomes and degraded by autophagosome-lysosome fusion in host cells. However, several pathogens, such as *Salmonella* and *Shigella*, avoid and/or escape autophagic degradation to establish infection. But autophagy involvement as a host immune response to *C. jejuni* infection has not been clarified. This study revealed autophagy association in *C. jejuni* infection. During infection, *C. jejuni* activated the Rho family small GTPase Rac1 signaling pathway, which modulates actin remodeling and promotes the internalization of this pathogen. In this study, we found the LC3 contribution to *C. jejuni* invasion signaling *via* the Rac1 signaling pathway. Interestingly, during *C. jejuni* invasion, LC3 was recruited to bacterial entry site depending on Rac1 GTPase activation just at the early step of the infection. *C. jejuni* infection induced LC3-II conversion, and autophagy induction facilitated *C. jejuni* internalization. Also, autophagy inhibition attenuated *C. jejuni* invasion step. Moreover, Rac1 recruited LC3 to the cellular membrane, activating the invasion of *C. jejuni*. Altogether, our findings provide insights into the new function of LC3 in bacterial invasion. We found the interaction between the Rho family small GTPase, Rac1, and autophagy-associated protein, LC3.

## Introduction

*Campylobacter jejuni* is a Gram-negative spiral bacterium, which causes food-borne disease and induces gastroenteritis symptoms. Genomic study revealed that *C. jejuni* does not conserve any significant toxin or virulent genes, such as type III secretion system (T3SS), widely possessed by Gram-negative pathogens (Parkhill et al., 2000). Alternatively, several studies have revealed the association of various factors in the pathogenicity of *C. jejuni*, such as motility, chemotaxis, and invasion (Golden and Acheson, 2002; Hendrixson, 2006). Additionally, *C. jejuni* mutant strain infection, which invade host cells, attenuate the production of proinflammatory cytokine interleukin-8 (IL-8) from intestinal epithelial cells (Hickey et al., 2000). So that, adhesion and invasion steps were considered the key factor of virulence in *C. jejuni* infection.

During *C. jejuni* infection, actin filament rearrangement, microtubule dynamics, and endocytosis pathway, were involved in the *C. jejuni* invasion step (Wooldridge et al., 1996; Biswas et al., 2003; Krause-Gruszczynska et al., 2007; Kido et al., 2017). Alternatively, bacterial fibronectin-binding proteins, CadF and FlpA, are regarded as one of the main bacterial adherence factors that activate integrins and trigger host cytoskeletal rearrangement signaling pathway, and facilitate bacterial internalization (Konkel et al., 1997; Talukdar et al., 2020). Furthermore, integrins α5β1 promote focal adhesion kinase (FAK) phosphorylation, which induces the activation of Rac1 GTPase and actin filament remodeling (Boehm et al., 2011; Eucker and Konkel, 2012). In *C. jejuni* infection, Rac1 GTPase activation enhanced the invasion of this pathogen (Boehm et al., 2011), so that it is regarded a crucial role in *C. jejuni* entry step into host epithelial cells.

The bacterial invasion triggers the host immune defense machinery and eliminates intracellular pathogens (Baxt et al., 2013). Particularly, autophagy, especially xenophagy, is characterized as an antibacterial degradation process (Yang and Klionsky, 2009; Deretic, 2011). Autophagy is highly conserved in eukaryotic cells and leads to the lysosomal degradation pathway (Levine et al., 2011). In this pathway, invading pathogens are captured into autophagosomes and degraded by lysosomal fusion. Indeed, in Group A streptococcus (GAS) infection, invading pathogens are eliminated selectively by xenophagy, so that autophagy induction limits GAS infection (Nakagawa et al., 2004). Alternatively, several pathogens, such as *Listeria monocytogenes* and *Shigella flexneri* have escaped systems from xenophagy to infect host cells effectively (Ogawa et al., 2005; Yoshikawa et al., 2009; Dortet et al., 2011). Additionally, it is reported that *Francisella tularensis* and *Brucella abortus* rather utilize autophagy signaling for bacterial survival (Checroun et al., 2006; Starr et al., 2012). However, it is still not entirely understood how the *C. jejuni* interacts with the host autophagy during infection.

It was unclear whether autophagy, which was activated by *C. jejuni* infection, acts as a host immune system or contributes to the infection process of this pathogen. Therefore, we investigated whether *C. jejuni* was trapped by autophagy organelles for degradation, and this bacterium did not colocalize with autophagosomes and autolysosomes, unlike *Salmonella* Enteritidis. This study revealed the significance of *C. jejuni*-induced autophagy, which did not work as an intracellular clearance system against this pathogen. Moreover, *C. jejuni*-induced autophagy participated in the invasion of this bacterium, which facilitated the infection. Here, we estimated the contribution of autophagy-related protein, LC3, to the classic *C. jejuni* invasion pathway, Rac1 signaling. *C. jejuni* invasion was facilitated by Rac1 activation, and it was canceled by autophagy inhibitor treatment. Additionally, LC3 was recruited to *C. jejuni* entry site depending on the Rac1 GTP activity. The recruitment of LC3 around *C. jejuni* was suppressed in the CadF mutant strain. Fibronectin-binding partner of *C. jejuni*, CadF activate Rac1 *via* host integrins-FAK signaling pathway to trigger actin rearrangement (Boehm et al., 2011; Talukdar et al., 2020). Those results suggest that the intracellular localization of LC3 was regulated *via* the FAK-Rac1 signaling pathway in *C. jejuni* infection (Boehm et al., 2011; Eucker and Konkel, 2012). Additionally, we indicated the molecular interaction between Rac1 and LC3 by immunoprecipitation. Thus, these results lead to a new role of LC3 in bacterial invasion.

## Results

### *C. jejuni* Induced Autophagy But Bacterial Cells Were Not Colocalized With Autophagy Organelles

**Figure 1:**
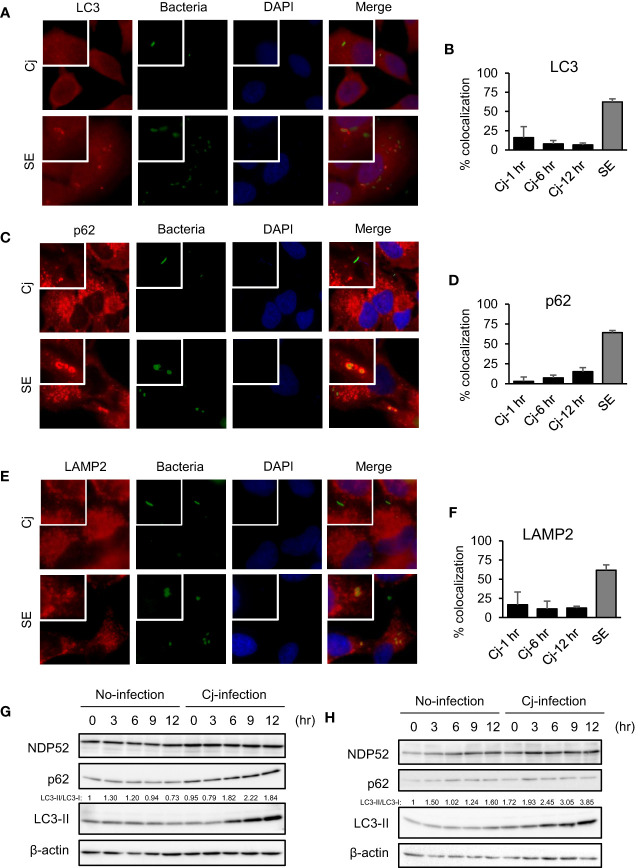
The indication of autophagy, and localization of autophagosomes/lysosomes and pathogens in *C. jejuni* infection. **(A, C, E)** Representative immunofluorescence images of HeLa cells infected with *S*. Enteritidis (SE) for 1 h or *C. jejuni* (Cj) for 6 h. *S*. Enteritidis and *C. jejuni* was stained with 10 ng/ml 5(6)-Carboxyfluorescein diacetate (CFDA) solution, and infected to HeLa cells. Cells were immunostained by anti-LC3 **(A)**, p62 **(C)**, and LAMP2 **(E)** antibody and DAPI, and observed by Keyence BZ-X700 fluorescence microscope. **(B)** Intracellular bacteria which co-localized with LC3 were quantified. Co-localization rate was determined at 1 h *p. i*. (Cj-1 h), 6 h *p. i*. (Cj-6 h), and 12 h *p. i*. (Cj-1 hr) in ***(C)*** *jejuni* infection, and 1 h *p. i*. (SE) in *S*. Enteritidis infection. Cj-1 h: 15.93 ± 14.33% of colocalization; n = 36, Cj-6 h: 7.88 ± 4.30% of colocalization; n = 55, Cj-12 h: 6.54 ± 2.38% of colocalization; n = 94, and SE: 62.40 ± 4.16% of colocalization; n = 186. Sample number indicated the sum of 3 independent experiments. **(D)** Intracellular bacteria which co-localized with p62 were quantified. Cj-1 h: 3.03 ± 5.25% of colocalization; n = 37, Cj-6 h: 7.35 ± 3.35% of colocalization; n = 59, Cj-12 h: 15.13 ± 5.05%; 12 h *p. i*. of colocalization; n = 117, and SE: 64.25 ± 2.50% of colocalization; n = 191. Sample number indicated the sum of 3 independent experiments. **(F)** Intracellular bacteria which co-localized with LAMP2 were quantified. Cj-1 h: 16.67 ± 16.67% of colocalization; n = 35, Cj-6 h: 11.21 ± 10.22% of colocalization; n = 55, Cj-12 h: 15.13 ± 5.05%; 12 h *p. i*. of colocalization; n = 96, and SE: (61.72 ± 6.99% of colocalization; n = 115. Sample number indicated the sum of 3 independent experiments. **(G)** HeLa cells were infected with *C. jejuni* NCTC 11168 up to 12 h and detected autophagy-related proteins, NDP52, p62/SQSTM, LC3 by Western blotting. **(H)**. Caco-2 cells were infected with *C. jejuni* and detected autophagy-related protein levels.

We observed the localization of autophagy organelles and intracellular bacteria to estimate whether *C. jejuni* are targeted by xenophagy as with *Salmonella* and *Shigella* infection (Kageyama et al., 2011; Tattoli et al., 2012). In xenophagy, adapter proteins, such as p62/SQSTM1 and NDP52, crosslinks between autophagy cargo and LC3 *via* LIR (LC3-interacting region) motif to lysosomal degradation (Birgisdottir et al., 2013; Sudhakar et al., 2019). In this experiment, *S*. Enteritidis was applied as a positive control of the target in xenophagy. Immunofluorescence staining revealed that most *S*. Enteritidis was colocalized with LC3 (62.40 ± 4.16% of colocalization, n = 186; **Figures 1A, B**) and p62 (64.25 ± 2.50% of colocalization, n = 191; **Figures 1C, D**), and these data suggest that *S*. Enteritidis was engulfed in autophagosomes (**Figures 1A**–**D**). Alternatively, *C. jejuni* were not colocalized with autophagosomal proteins, LC3 (15.93 ± 14.33%, n = 36; 1 h *p.i.*, 7.88 ± 4.30%, n = 55; 6 h *p.i.*, and 6.54 ± 2.38%, n = 94; 12 h *p. i*., n = 94) and p62 (3.03 ± 5.25%, n = 37; 1 h *p.i.*, 7.35 ± 3.35%, n = 59; 6 h *p.i.*, and 15.13 ± 5.05%; 12 h *p. i*., n = 117), respectively (**Figures 1A**–**D**). Also, *C. jejuni* were not targeted by NDP52 (**Figures S1A, B**). Autophagosomes mature into autolysosomes by fusing with lysosomes in the late stage of autophagy flux to degrade contents of the vesicles. Thus, we checked the lysosomal localization in *C. jejuni*-infected cells. But, *C. jejuni* indicated lower colocalization with lysosomal membrane protein, LAMP2 (16.67 ± 16.67%, n = 35; 1 h *p.i.*, 11.21 ± 10.22%, n = 55; 6 h *p.i.*, 12.42 ± 2.14%, n = 96; 12 h *p. i*.) compared with *S*. Enteritidis (61.72 ± 6.99%, n = 115, **Figures 1E, F**). Similar results were obtained in another *C. jejuni* strain, 81−176, infected cells (**Figures S1C**–**H**). Next, we checked autophagy induction in *C. jejuni*-infected cells by Western blotting. As shown in **Figures 1G, H**, LC3-II levels, the marker of autophagosome production, was increased during 6–12 h in *C. jejuni* (NCTC11168) infection in HeLa and Caco-2 cells. Still, autophagy adaptor proteins, p62 and NDP52, were unchanged. Many other autophagy-related (ATG) proteins were also detected, but their protein levels were unchanged, same as p62 and NDP52 during *C. jejuni* infection (**Figures 1G, H** and **Figure S2A**). Also, the other *C. jejuni* strains 81−176, and 81116 infected cells indicated similar results (**Figures S2B, C**). To confirm that LC3 increase was associated with autophagy induction, we monitored autophagy flux. Consequently, Bafilomycin A1 treatment increased LC3-II level with the increasing of LC3B mRNA expression in *C. jejuni* infection (**Figures S2D**–**F**). Additionally, colocalization of LC3 and LAMP2 was increased (**Figures S2G, H**). These data indicated that autophagy flux was not inhibited in *C. jejuni* infection, and implied that *C. jejuni* upregulated autophagy induction. Altogether, these data revealed that *C. jejuni* infection triggers autophagy, but this pathogen was not targeted by autophagosomal or autolysosomal compartments.

### Autophagy is Involved in *C. jejuni* Invasion

**Figure 2:**
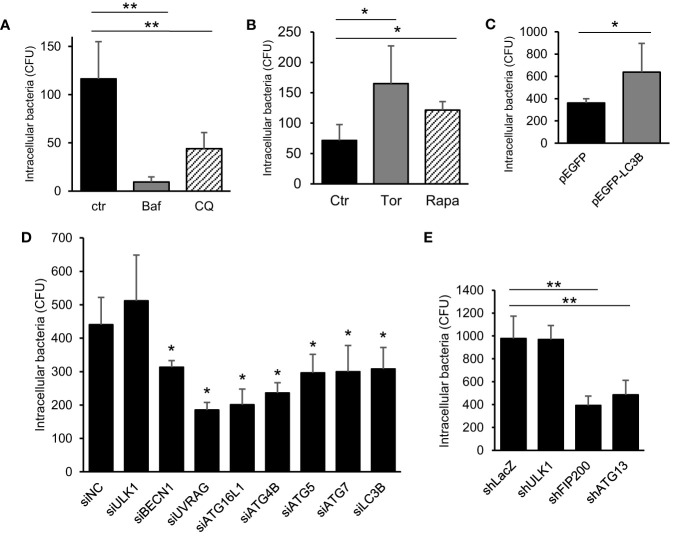
The contribution of autophagy signaling in *C. jejuni* invasion step. **(A)** HeLa cells were treated with 200 nM Bafilomicin-A1 (Baf) or 50 μM Chloroquine (CQ) for 3 h. The number of intracellular *C. jejuni* was measured by gentamicin protection assay at 1 h *p. i*., and decided by counting of Colony Forming Unit (CFU). **(B)** The number of intracellular *C. jejuni* in HeLa cells, which treated by 1 μM Torin 1 (Torin) or 200 nM Rapamycin (Rapa) for 3 h, were measured by gentamicin protection assay at 1 h *p. i*. **(C)** HeLa cells which stably express pEGFP or pEGFP-LC3B vector were infected with *C. jejuni* for 1 h, and the number of invaded bacteria was estimated by gentamicin protection assay. **(D)** siRNA against autophagy-related genes were transfected to HeLa cells, and intracellular bacterial number at 1 h *p. i*. was measured by gentamicin protection assay. **(E)** The expression of ULK1 complex components, ULK1, FIP200, and ATG13, were attenuated by shRNA-mediated knockdown in HeLa cells, and the invaded bacterial number was measured by gentamicin protection assay at 1 h *p. i*. All data are expressed as means ± standard deviations from 3 independent experiments. Differences were evaluated with Two-tailed Student’s t-test. *p < 0.05, **p < 0.001.

We explored the atypical role of autophagy induced by *C. jejuni*. As shown in **Figure 1**, autophagy was not associated with *C. jejuni* clearance in host cells. Next, we focused on bacterial invasion with earlier steps of infection. Intracellular bacteria were decreased in autophagy inhibitors, Bafilomycin A1 (Baf) and Chloroquine (CQ), treated cells (**Figure 2A**). Also, treatment of autophagy inducer, Torin 1 (Tor) and Rapamycin (Rapa), facilitated *C. jejuni* invasion in HeLa cells (**Figure 2B**). Similar results were observed in the intestinal epithelial cell line, Caco-2 cell (**Figures S3A, B**). Autophagy involvement in *C. jejuni* invasion step was confirmed using the overexpression of autophagosome component (**Figure 2C**) and knockdown of siRNA or shRNA-mediated autophagy genes (**Figures 2D, E**). The bacterial invasion was increased in exogenous LC3-II expressing cells (**Figure 2C** and **Figure S4A**). Additionally, knockdown of many components, which compose the autophagy pathway, reduced the entry of *C. jejuni* into host cells (**Figures 2D, E**). On the other hand, invasion step of *S*. Enteritidis was not changed in autophagy inducers or knockdown of siRNA-mediated autophagy genes (**Figures S3D, E**). Interference efficiency of siRNA and shRNA was confirmed by Western blotting (**Figures S4B**–**M**). Interestingly, siRNA-mediated ULK1 knockdown, which participates in autophagosome nucleation, did not affect invasion (**Figure 2D**). ULK1 plays a central role in autophagy induction by forming a complex with ATG101, FIP200, and ATG13 (Mercer et al., 2009; Karanasios et al., 2016). Thus, we confirmed the participation of other factors of ULK1 complex, FIP200, and ATG13 in *C. jejuni* invasion with shRNA. Opposite to ULK1, FIP200 and ATG13 were involved in the bacterial invasion step (**Figure 2E**).

Altogether, these data suggest that most autophagy components were associated with *C. jejuni* invasion, and autophagy could be used in the bacterial invasion during infection to host epithelial cells; new autophagy function.

### Association Between Autophagy- and Rac1-Mediated *C. jejuni* Invasion Pathways

**Figure 3:**
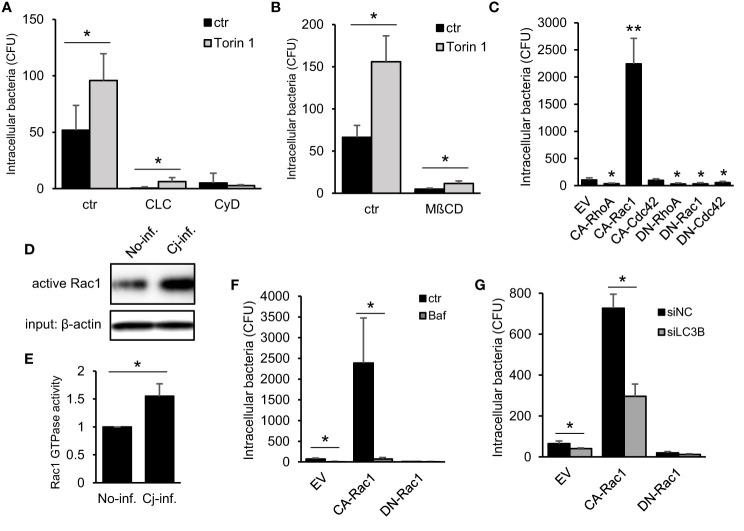
Association between Autophagy- and Rac1-mediated *C. jejuni* invasion pathway. **(A)** HeLa cells were treated with 10 μM Cholchicine (CLC), 300 nM Cytochalasin D (CyD) with or without 1 μM Torin 1 for 3 h. The number of invaded *C. jejuni* was estimated by gentamicin protection assay at 1 h *p. i*. **(B)** HeLa cells were treated with 1 μM Torin 1 for 3 h, and 3 mM Methyl-beta-cyclodextrin (MβCD) was additionally treated for last 1 h. Intracellular bacterial number was measured by gentamicin protection assay at 1 h *p. i*. **(C)** Empty vector (EV) and, constitutive active form (CA) and dominant negative form (DN) of RhoA, Rac1, and Cdc42 were transfected in HeLa cells. The number of intracellular *C. jejuni* was estimated by gentamicin protection assay at 1 h *p. i*. **(D)** Intracellular active Rac1 level at 6 h *p. i*. was measured by Western blotting. **(E)** Band intensity of active Rac1 was quantified. **(F)** CA-Rac1 or DN-Rac1 expressed HeLa cells were treated with 200 nM Bafilomycin A1 (Baf) for 3 h. The number of intracellular *C. jejuni* was estimated by gentamicin protection assay at 1 h *p. i*. **(G)** HeLa cells were co-transfected EV, CA-Rac1, or DN-Rac1 and siNC or siLC3B. The number of intracellular *C. jejuni* was estimated by gentamicin protection assay at 1 h *p. i*. All data are expressed as means ± standard deviations from 3 independent experiments. Differences were evaluated with Two-tailed Student’s t-test. *p < 0.05, **p < 0.001.

Next, we undertook to clarify the mechanism of how autophagy signaling promotes *C. jejuni* invasion. Since the association between autophagy and bacterial invasion signaling is not clearly understood. Recent studies characterized that *C. jejuni* invasion is associated with cytoskeletal dynamics, such as microtubules (Hu and Kopecko, 1999; Kido et al., 2017) or actin filaments (Biswas et al., 2003; Krause-Gruszczynska et al., 2007), and intracellular transportation pathway, endocytosis (Wooldridge et al., 1996). We focused on autophagy association in conventional invasion signaling in *C. jejuni*. To assess the effect of autophagy signaling to classical invasion signaling in *C. jejuni* invasion, the cells were treated with each signaling inhibitor: colchicine, cytochalasin D, or MβCD, in addition to with/without the autophagy inducer, Torin 1. Consequently, each inhibitor attenuated bacterial invasion, and Torin 1 treatment promoted the bacterial invasion even microtubule dynamics and endocytosis inhibitors-treated cells. Alternatively, Torin 1-mediated increase in *C. jejuni* invasion was abolished in obstruction of actin assembly by cytochalasin D treatment (**Figures 3A, B**). These data indicated that autophagy-mediated *C. jejuni* invasion was associated with actin assembly signaling. Since the actin assembly was modulated by several types of small GTPases, we next surveyed the contribution of small GTPases that drive actin dynamics. Here we applied constitutively active form or dominant-negative form of Flag-tagged RhoA, Rac1, and Cdc42 expressed in cells (**Figure S5A**). *C. jejuni* invasion was dramatically increased in active Rac1-expressed cells (**Figures 3C**–**E** and **Figure S5B**: 81−176 strain). Also, *C. jejuni* infection increased endogenous Rac1 activity, the same as in previous reports (Krause-Gruszczynska et al., 2007; Eucker and Konkel, 2012). Additionally, the increase in *C. jejuni* invasion by Constitutive active (CA)-Rac1 was canceled by the autophagy inhibitor, Bafilomycin A1 treatment (**Figure 3F**). It was also confirmed by using LC3B knockdown by siRNA (**Figure 3G**). These results indicate that the actin polymerization factor, Rac1, was involved in the autophagy-mediated *C. jejuni* invasion step. Additionally, actin polymerization or Rac1 signaling was nonessential for *C. jejuni* adhesion step (**Figure S5F**). Alternatively, Rac1-associated uptake was not found in *Salmonella* (*S*. Enteritidis and *S*. Typhimurium) invasion and uptake of FITC-dextran (**Figures S5C**–(**E**). Therefore, of Rac1 contribution was specific to *C. jejuni* invasion.

### Interaction Between Autophagy and Rac1 Signaling in *C. jejuni* Infection

**Figure 4:**
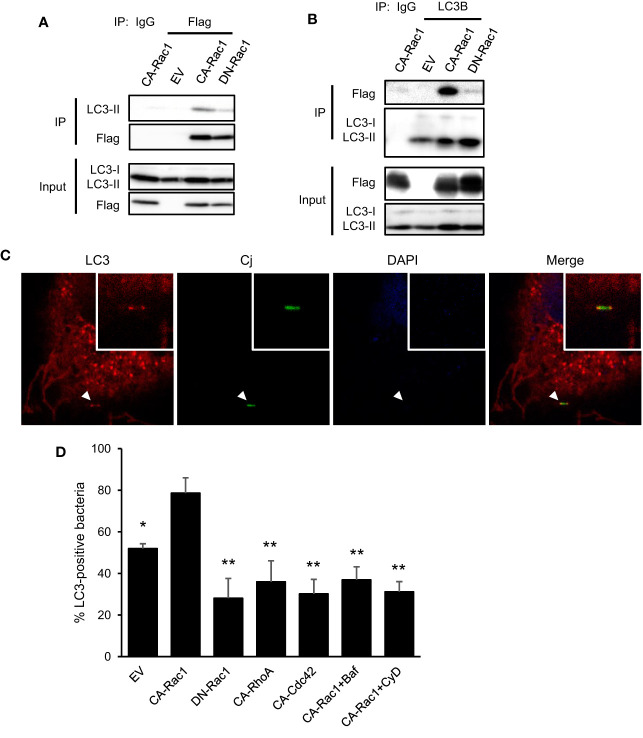
Interaction of autophagy signaling and Rac1 signaling in *C. jejuni* infection. **(A)** HeLa cells were transfected with EV, CA-Rac1, and DN-Rac1. Cells were lysed and subjected to immunoprecipitation with Sepharose beads, and analyzed by Western blotting by using anti-Flag antibody. **(B)** HeLa cells were transfected with vectors as shown, and subjected to immunoprecipitation. Cell lysates were analyzed by Western blotting by using anti-LC3B antibody. **(C)** Representative immunofluorescence images of CA-Rac1 expressed HeLa cells infected with *C. jejuni* which stained with 10 ng/ml 5(6)-Carboxyfluorescein diacetate (CFDA) solution. Cells were immunostained by anti-LC3 antibody and DAPI, and observed by confocal microscope. **(D)** The rate of colocalization of *C. jejuni* and LC3 was quantified in EV, CA-Rac1, DN-Rac1, CA-RhoA, and CA-Cdc42 expressed HeLa cells. CA-Rac1 transfected cells were treated with 200 nM Bafilomycin A1 (Baf) for 3 h or 300 nM Cytochalasin D (CyD) for 1 h before infection. The differences of signnificance were tested in comparison with CA-Rac1. EV: n = 48, CA-Rac1: n = 73, DN-Rac1: n = 59, CA-RhoA: n = 65, CA-Cdc42: n = 83, CA-Rac1 + Baf: n = 55, CA-Rac1 + CyD: n = 51 of bacterial cells. The data of confocal microscope were pooled from 4 independent experiments. Differences were evaluated with Two-tailed Student’s t-test. *p < 0.05, **p < 0.001.

We investigated the interaction between autophagy and Rac1 in *C. jejuni* infection to promote bacterial entry. First, the molecular interaction between autophagy-associated protein and Rac1 was confirmed, immune precipitation experiment revealed LC3 binding to Flag-Rac1 (**Figures 4A, B**). This interaction was clearly enhanced in CA-Rac1, and it is indicated that Rac1 regulates binding to LC3 depending on the GTPase activity. Additionally, LC3 was colocalized with CA-Rac1 and recruited this protein to the edge of the cell (**Figures 4C, D**; EV: 51.97 ± 2.33%, n = 48, CA-Rac1: 78.58 ± 7.38%, n = 73, DN-Rac1: 28.08 ± 9.52%, n = 59, CA-RhoA: 35.96 ± 10.12%, n = 65, CA-Cdc42: 30.20 ± 6.92%, n = 83, CA-Rac1 + Baf: 36.89 ± 6.28%, n = 55, CA-Rac1 + CyD: 31.17 ± 4.87%, n = 51). Moreover, *C. jejuni* was colocalized with LC3 at the bacterial entry site only in the early stage of infection, contrary to observations during its intracellular survival (**Figures 1A, B** and **4C**). The percentage of *C. jejuni* colocalized with LC3 was increased in CA-Rac1 and decreased in DN-Rac1 expressed cells. Other small GTPases-expressed cells or autophagy and actin polymerization inhibitor-treated CA-Rac1 expressed cells decreased colocalization with LC3 (**Figure 4D**). This change in the recruitment of LC3 to *C. jejuni* invasion sites was consistent with promoted or attenuated bacterial invasion (**Figures 3C** and **4B, C**). Unlike LC3, autophagy adaptor protein, p62, was not colocalized with bacteria. Those results showed the characteristic recruitment of LC3 in *C. jejuni* infection, and that mechanism was different from xenophagy (**Figure S6A**). Our results indicated that LC3 recruitment to the *C. jejuni* invasion site was driven by a direct interaction between LC3 and Rac1, and LC3 was used in *C. jejuni* invasion *via* Rac1 signaling pathway.

### Activation Pathway of Rac1 Signaling That Recruits LC3 to *C. jejuni* Entry Site

**Figure 5:**
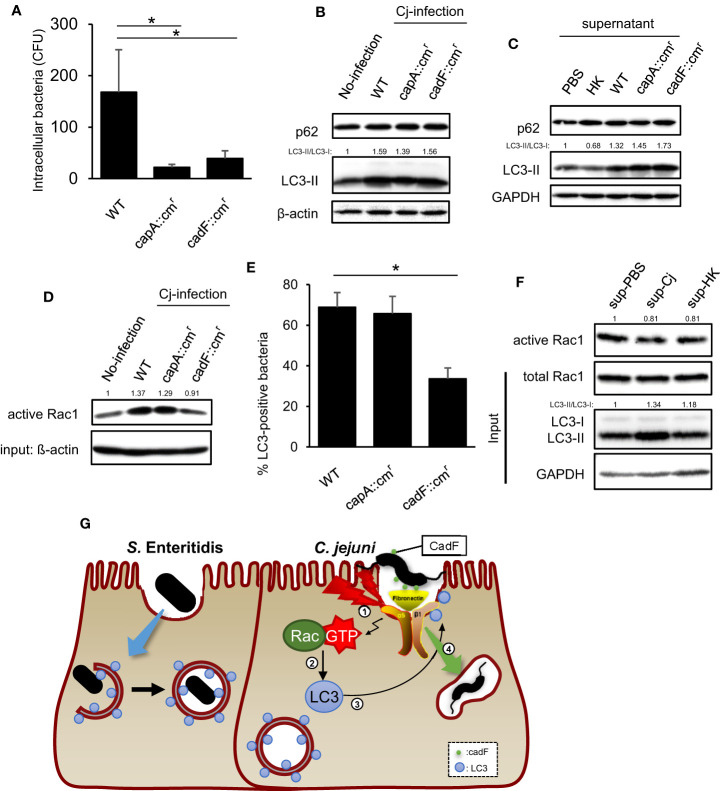
Contribution of virulence factor in LC3-mediated *C. jejuni* invasion. **(A)** HeLa cells were infected with *C. jejuni* of WT, capA::cm^r^, and cadF::cm^r^. The number of intracellular *C. jejuni* was estimated by gentamicin protection assay at 1 h *p. i*. Data are expressed as means ± standard deviations (n = 4). **(B)** HeLa cells were infected with *C. jejuni* for 6 h, and detected autophagy-related proteins, p62/SQSTM and LC3 by Western blotting. **(C)** *C. jejuni* strains of WT, capA::cm^r^, and cadF::cm^r^ or heat-killed bacteria were incubated in High glucose supplemented DMEM [DMEM(+)] for 24 h, and then bacterial cells were removed and the supernatant was collected. HeLa cells were cultured in bacterial removed supernatant for 6 h, and detected p62/SQSTM and LC3 by Western blotting. **(D)** Intracellular active Rac1 level at 6 h *p. i*. was measured by Western blotting. **(E)** Localization of LC3 and *C. jejuni* was observed by confocal microscope at 20 min *p.i.*, and the rate of LC3-positive bacteria was quantified in *C. jejuni* of WT, capA::cm^r,^ and cadF::cm^r^ infected cells. WT: n = 64, capA::cm^r^: n = 83, cadFcm^r^: n = 84 of bacterial cells which pooled from 3 independent experiments. **(F)** *C. jejuni* and heat-killed bacteria was incubated in DMEM(+) for 24 h, and the supernatant was collected. HeLa cells were cultured in aforementioned supernatant for 6 h, and intracellular active Rac1 level was measured by Western blotting. **(G)** Proposed model in this study for *C. jejuni* infection signaling which mediated by Rac1 activated by CadF, and invasion route *via* LC3. All data are expressed as means ± standard deviations from 3 independent experiments. Differences were evaluated with Two-tailed Student’s t-test. *p < 0.05.

Finally, we explored what signaling pathway activates Rac1 and recruits LC3 to internalize bacteria. A previous study revealed that *C. jejuni* fibronectin-binding partner, CadF, activated Rac1 *via* host integrins-FAK signaling to trigger actin rearrangement (Boehm et al., 2011; Talukdar et al., 2020). We investigated the correlation between the Rac1 signaling pathway above, and the other is the Rac1 activation pathway to alter the intracellular localization of LC3 and classical *C. jejuni* invasion-related Rac1 signaling. Two invasion deficient strains, CadF::cm^r^ and CapA::cm^r^ were applied in this study (**Figure 5A**). Although, the reduction of intracellular entry, LC3-II level was increased in each *C. jejuni* strain (**Figure 5B**). Additionally, bacterial removed supernatant, including bacterial secretion, indicated the autophagy induction ability (**Figure 5C**). These data suggest that autophagy was induced by *C. jejuni* secretion, independently of invasion. Unlike intracellular LC3-II level, only CadF mutant did not activate host Rac1 signaling (**Figure 5D**). Interestingly, recruitment of LC3 to bacterial entry site was decreased in CadF mutant, and it was implied that *C. jejuni* recruit LC3 to bacterial invasion site by binding host fibronectin to activate classical *C. jejuni* invasion signaling, the Rac1 signaling pathway (**Figure 5E**). Additionally, supernatant induced autophagy but it did not activate Rac1 (**Figure 5F**). Our data suggest that the Rac1 signaling pathway was triggered by CadF-mediated integrin-FAK signaling, and it regulates the localization of LC3 to the bacterial entry site (**Figure 5G**).

## Discussion

In this study, we clarified the new function of autophagy in *Campylobacter jejuni* infection. In bacterial infection, autophagy is characterized as an intracellular catabolic system to maintain homeostasis, and it degrades invading pathogens as the host immune defense system. However, *C. jejuni* is not colocalized with LC3, p62, and NDP52 (**Figures 1A**–**H**), which means that *C. jejuni* was not targeted by the xenophagy within the cell. Alternatively, autophagosome protein, LC3-II, significantly increased in infected cells. The accumulation of autophagy protein comes from the upregulation of the pathway or inhibition of autophagy flux (Mizushima and Yoshimori, 2007). In this study, the LC3-II level was more increased by autophagy inhibitor treatment, Bafilomycin A1, in *C. jejuni* infection (**Figures 1L, M**). Additionally, mRNA expression of LC3B was upregulated by *C. jejuni* infection (**Figure 1N**). These results suggest that *C. jejuni* activated autophagy signaling rather than inhibiting it. Additionally, autophagy inhibition did not recover the survival ratio of intracellular *C. jejuni* (Data not shown). These results indicate that autophagy induced by *C. jejuni* infection does not work to eliminate this pathogen.

As another function of autophagy that does not remove intracellular pathogens, autophagy utilization in the invasion step of *C. jejuni* was newly discovered in this study. Recently, it has been reported that autophagy proteins are involved in the cellular uptake pathway *via* an atypical autophagy signaling pathway. LC3-associated phagocytosis (LAP) is one of the noncanonical autophagy pathways in which LC3 is recruited to mature phagosomes and enhances the immune response (Martinez et al., 2011). Furthermore, LC3-associated with endocytosis (LANDO) is involved in the uptake and degradation of amyloid β in nerve cells (Heckmann et al., 2020). Those pathways newly revealed intracellular uptake pathway *via* noncanonical autophagic vesicle, which recruits LC3. However, autophagy-mediated *C. jejuni* invasion pathway was considered as different in the following points: (i) Differences in participation of autophagic components involved in the signaling pathway; Rubicon is a negative regulator of canonical autophagy whereas involved in noncanonical autophagy (Martinez et al., 2015). However, RNAi of Rubicon did not attenuate *C. jejuni* internalization; rather it increased (**Figure S2A**). Furthermore, the components of the ULK1 complex, ATG13 and FIP200, were involved in *C. jejuni* invasion, different from LAP and LANDO (**Figure 2E**). Briefly, autophagy signaling-mediated *C. jejuni* invasion differs from reported noncanonical autophagy in terms of Rubicon and ULK1 complex involvement. (ii) Difference in the time point of LC3 recruitment to vesicles; LC3 was localized to the maturated vesicles after the internalization of pathogens in the noncanonical autophagy pathway (Martinez et al., 2011; Heckmann et al., 2020), but in *C. jejuni* infection, LC3 was recruited to the bacterial invasion site, which is the initial stage of *Campylobacter*-containing vacuole (CCV) formation (**Figures 4C, D**). Afterward, the maturated CCV in the cytoplasm was changed to LC3 negative (**Figures 1A**–**F**), LC3 contributed to bacterial invasion, but was unrelated to CCV maturation. Namely, in *C. jejuni* infection, LC3 did not participate in CCV maturation, unlike other noncanonical autophagy. (iii) Association of Rac1 with noncanonical autophagy signaling pathway; no study indicated the contribution of Rac1 signaling for LC3 recruitment in noncanonical autophagy. Our data indicated that Rac1 was involved in the recruitment of LC3 to internalizing pathway of *C. jejuni*. Our observations indicated that autophagy signaling activated in *C. jejuni* infection deviated away from canonical autophagy. It is also different from known atypical autophagy pathway, such as LAP and LANDO. Thus, we provide new insights that autophagy induced by *C. jejuni* is involved in pathogen infection.

To identify the mechanism of autophagy-associated *C. jejuni* invasion, we used several types of inhibitors or small GTPase mutants. In this study, we identified Rac1, a small GTPase involved in actin polymerization, as a contributing factor for the invasion of *C. jejuni via* autophagy signaling (**Figure 3**). Additionally, we found that the interaction between LC3 and Rac1 enhanced its interaction by Rac1 activation. Furthermore, LC3 recruitment to the cell surface was controlled by Rac1 activation, which positively regulated *C. jejuni* invasion.

To clarify the bacterial factors, which regulate autophagy-mediated invasion, we used CapA and CadF mutant strains. CapA, an auto transporter protein of *C. jejuni*, is one of the factors that contribute to internalization in host cells (Ashgar et al., 2007). Alternatively, the outer membrane fibronectin-binding protein, CadF, mediates adhesion to cell-associated fibronectin and activates Rac1, which is mediated by Paxillin phosphorylation (Eucker and Konkel, 2012). This signaling pathway contributes to *C. jejuni* internalization by acactivating actin polymerization. Consistent with the role of CapA and CadF in the infection, mutant strains of these genes attenuated bacterial invasion. Additionally, the CadF mutant strain reduced Rac1 activation and colocalization with LC3 and decreased intracellular bacteria. In contrast, CapA::cm^r^ reduced invasion, but Rac1 activity and LC3 colocalization were the same as WT (**Figures 5A, D, E**). Those results indicate that *C. jejuni* internalization pathway mediated by Rac1-LC3 signaling axis was activated by *C. jejuni* outer membrane protein, CadF. Interestingly, Rac1 activation level was different between wildtype and these two mutants, but LC3 protein level was the same in each strain-infected cell (**Figure 5B**). This result indicated that the activation of autophagy signaling was upregulated by *C. jejuni* infection independent of CadF-Rac1 signaling. It suggested that *C. jejuni* facilitated invasion *via* the dual pathway of autophagy induction and LC3 localization. The addition of bacterial-cultured supernatants, which removed bacterial cells, also increased LC3 and the infection of living *C. jejuni*, this data suggested that this pathogen-induced autophagy occurs in the earliest step of the infection before Rac1 signaling activation (**Figure 5C**). In other words, *C. jejuni* enhanced the LC3-II protein level from outside the cell, and could change the host condition to be specialized for establishing the infection. Then, adhered pathogen recruited increased LC3 to bacterial entry site by activating Rac1, and efficiently invaded the host cells. These led to the conclusion that an autophagy-mediated invasion is one of the infection pathways for *C. jejuni*. Additionally, we also found the new function of Rac1 in the noncanonical autophagy pathway that concerns relocalization of LC3 distribution in *C. jejuni* infection.

In this study, it is insufficiently understood how Rac1 interacts with the autophagy signaling pathway. Autophagy has roles in starvation adaptation, intracellular protein clearance, and cell death *via* the mTORC1 signaling pathway (Mizushima, 2007). Autophagy induction is closely associated with mTORC1 signaling, and under autophagy induced conditions, downstream factors of mTORC1 are dephosphorylated (Jung et al., 2010). Previously, some researchers have found mTORC1 regulation and autophagy signaling by Rac1, but there are inconsistent insights into how Rac1 positively and negatively controls autophagy (Xie et al., 2018; Ma et al., 2019; Zhang et al., 2020). These different results could be due to the role of Rac1 in intracellular processes, and autophagy regulation is changed depending on various cellular conditions and neoplasticity. In our results, CA and DN overexpression in several types of small GTPase, including Rac1, modulated p70S6K to be phosphorylated, which inhibits autophagy induction. However, in *C. jejuni*-infected cells, small GTPase-expressed cells decreased phosphorylated p70S6K and increased LC3-II production in *C. jejuni* infection (**Figure S6E**). It was suggested that the control of autophagy signaling by *C. jejuni* infection is more influential than that by Rac1 signaling. Here, we inadequately revealed the interaction between mTORC1-mediated autophagy induction and CadF-Rac1 mediated recruitment of LC3 to bacterial entry site. To clarify the interaction between the two signaling pathway, further examinations are required in the next study.

Usually, autophagy eliminates intracellular bacteria, but several pathogens use it to maintain bacterial survival in intracellular niches. Previously, several studies have revealed that intracellular pathogens, *F. tularensis* and *B. abortus*, partially recruit autophagy-related proteins to intracellular bacteria-containing vesicles (BCV) (Checroun et al., 2006; Starr et al., 2012; Steele et al., 2013). The maturation of BCV *via* a noncanonical autophagy signaling pathway contributes to bacterial intracellular survival. Additionally, it was suggested that the degradation of cellular contents by autophagy supply the nutrients required for intracellular survival of *F. tularensis* (Steele et al., 2013). Those studies indicated insights that lead to the control of pathogen infection by elucidating noncanonical autophagy signaling pathway in bacterial infection. Similarly, here we indicated new insights of noncanonical autophagy-mediated *C. jejuni* invasion pathway. Altogether, it is suggested that autophagy may have a potential for preventing and inhibiting major foodborne-causing pathogens, *C. jejuni* infection. To clarify the therapeutic potential, a detailed pathway of intracellular invasion mediated by LC3 is necessary for future studies.

## Materials and Methods

### Bacterial Strains and Culture Conditions

*C. jejuni* strains NCTC11168 (ATCC 700819), 81-176 (ATCC BAA2151), and 81116 (NCTC11828) were obtained from the American Type Culture Collection (ATCC). The bacteria were grown in Muller–Hinton (MH) broth (Difco, #225250) at 37°C under micro aerobic conditions (5% O_2_, 10% CO_2_, 85% N_2_) for 48 h. It was concentrated by centrifugation at 12,000 rpm for 3 min, and bacterial pellet was diluted into fresh MH broth and was re-cultured under micro aerobic condition for 36 h. Then, bacterial cells were collected by centrifuging the medium at 3,000 rpm for 15 min, the supernatant was removed, and the pellet was washed with phosphate-buffered saline (PBS; 137-mM NaCl, 8.1-mM anhydrous Na_2_HPO_4_, 2.68-mM KCl, 1.47-mM KH_2_PO_4_). Then, suspension was centrifuged again, and the pellet was resuspended in PBS to be adjusted to an optical density of 600 nm (OD_600_) of 1.0 for the infection experiment.

*S. enterica* serovar Enteritidis (*S*. Enteritidis) 171 strain (Nakano et al., 2012) was cultured in Luria–Bertani (LB) medium at 37°C with shaking. For the experiment, bacterial cells were centrifuged at 12,000 rpm for 3 min, pellet were washed with PBS, centrifuged again, and resuspended in PBS to adjust to OD_600_ of 1.0.

### Cell Culture

HeLa cell (RCB #0007) was provided by the RIKEN BRC through the National BioResource Project of the MEXT/AMED, Japan, and cultured for three days in Dulbecco’s-modified Eagle’s medium, high glucose (DMEM; Sigma-Aldrich, #D6429) supplemented with 10% fetal bovine serum (FBS; Ausgene) and 50-µg/ml gentamicin (Wako, #078-06061) [DMEM (+)] at 37°C in 5% CO_2_. Additionally, the human intestinal epithelial cell line, Caco-2 cells, was cultured in DMEM (+) for four days.

For the infection experiments, the medium was changed to DMEM without FBS and gentamicin [DMEM (−)]. The cells were infected at a multiplicity of infection of 100−200:1 in *C. jejuni* infection or 10−20:1 in *S*. Enteritidis infection, and infected cells were incubated at 37°C in 5% CO_2_.

### Western Blotting

Cell lysates were prepared with Radio-Immunoprecipitation Assay buffer (RIPA buffer; pH 7.4, 50-mM Tris–HCl, 150 mM NaCl, 1-mM EDTA, 1% sodium deoxycholate, 0.1% SDS, 1% Triton X-100) mixed with 1-mM phenylmethanesulfonyl fluoride (PMSF) as a protease inhibitor. The cell lysates were centrifuged at 15,000 rpm for 10 min at 4°C, and the supernatant was collected in new tubes. Protein concentration was assayed using the BCA Protein Assay Kit (Wako, #297-73101). The protein was separated by 8–14% SDS-PAGE gel and transferred onto PVDF (Immunobilon-P; Millipore), and the membranes were blocked with 3% skim milk in TBS-T [pH 7.6, 20-mM Tris, 150-mM NaCl, 0.02% polyoxyethylene (20) sorbitan monolaurate] for 1 h at room temperature. After incubation, the membrane was treated with primary antibodies for LC3 (MBL, #PM036), p62 (MBL, #M162-3), NDP52 (GeneTex, #GTX115378), and ß-actin (SantaCruz, #sc-47778) at 1:1,000 dilution, and incubated at 4°C overnight. Afterward, the membrane was washed with TBST and treated with mouse monoclonal (MBL, #330) or rabbit polyclonal (MBL, #458) secondary antibodies (1:2,000) at room temperature for 90 min. The membranes were washed with TBS-T for 30 min and detected by enhanced chemiluminescence (ECL; Cytiva, #RPN2209) using medical X-ray film or a luminescent image analyzer (Fujifilm, LAS-2000).

### Immunofluorescence Staining

Before infection, the bacterial cells were incubated with 20-µM CFDA SE cell trace kit reagent (CFDA; Invitrogen, #V12883) dissolved in PBS for 1 h. Then, the bacterial suspension was centrifuged at 3,000 rpm for 15 min. Afterward; the pellets were washed with PBS, centrifuged again, and resolved in PBS to the infection experiment. HeLa cells were seeded on glass coverslips and infected with bacteria previously stained by CFDA.

The cells were fixed in 4% paraformaldehyde-PBS for 10 min at room temperature, permeabilized by 0.1% Triton X-100 solved in PBS for 7 min, and washed thrice with PBS. Next, samples were blocked with 3% BSA in PBS for 1 h, washed with PBS, and incubated with primary antibodies for LC3, p62, and LAMP2 (Abcam, #ab25631) at 1:500 dilution at 4°C overnight. Next, the samples were washed thrice with PBS and treated with secondary antibodies [Alexa Fluor^®^ 568-conjugated anti-mouse IgG (Invitrogen, #A11004) or Alexa Fluor^®^ 568-conjugated anti-rabbit IgG (Invitrogen, #A11011), 1:1,000] at 4°C for 1 h. Then, the samples were washed, and the cell nucleus was stained by 4’,6-Diamidino-2-Phenylindole, Dihydrochloride (DAPI; 500 nM, Invitrogen, #D1306) at 4°C for 5 min. Images were captured using a Keyence BZ-X700 microscope and Nikon A1R confocal laser scanning microscope.

### Plasmid

CA forms or dominant-negative (DN) forms of RhoA, Rac1, Cdc42 were subcloned into pME18sf-Pyori in a previous study (Akeda et al., 2002). All CA forms (RhoA^V14^, Rac1^V12^, and Cdc42^V12^) and DN forms (RhoA^N19^, Rac1^N17^, and Cdc42^N17^) contain N-terminal Flag tag.

LC3B overexpression plasmid (pEGFP-LC3B) was constructed from pEGFFP-C1, and the insert was created using the following primers; 5’-GAATTCGCCGTCGGAGAAGACCTTCAAGCAG-3’ for forward and 5’-GGATCCTTACACTGACAATTTCAATCCCGAACGTC-3’ for reverse.

### Extraction of *C. jejuni* Secretion Into the Culture Medium

*C. jejuni* were incubated in DMEM (−) overnight and the bacterial cells were removed using a syringe filter (Corning, #431215). After that, the flow through was ultra filtered using an Amicon Ultra 3K device (Merck Millipore, #UFC800324) at 3,000×*g* for 60 min. The culture medium on HeLa cells were replaced with the ultrafiltered flow through and evaluated autophagy induction.

### Invasion Assay

To conduct invasion assay, HeLa cells were seeded at a density of 5 × 10^4^ cells/well in 24-well plates and incubated at 37°C for three days.

In *C. jejuni* invasion assay, cells were treated with autophagy inhibitors, 200 nM Bafilomicin A1 or 50 µM Chloroquine, for 1 h before infection. The culture medium was replaced with flesh DMEM(−), and the cells were infected with *C. jejuni* for 1 h. Autophagy inducers, 1 μM Torin 1 or 200 nM Rapamycin, were incubated with cells for 1 h and the cells were infected with *C. jejuni* for 1 h. After infection, extracellular bacteria were removed and replaced the culture medium to 100 µg/ml gentamicin containing DMEM for 2 h. Then, the cells were washed with PBS and lysed with 1% Toriton-X in PBS for 5 min at 37°C. The diluted cell lysates were plated on MH agar plates and incubated for 48 h under micro aerobic conditions. Intracellular bacterial cell numbers were determined by counting colony-forming units (CFU), and the bacterial number was normalized with the protein concentration of the individual samples. Protein concentration was measured using BCA Protein Assay Kit (Thermo Fisher).

### Transfection With siRNA and shRNA

HeLa cells were seeded at a density of 1 × 10^5^ cells/well in 24-well plates and incubated for 24 h in DMEM(+). After that, the cell culture medium was removed and replaced with Opti-MEM (Thermo Fisher, #31985070). For siRNA-mediated knockdown, HeLa cells were transfected using Lipofectamine 2000 (Thermo Fisher, # 11668019) and predesigned siRNA for ULK1 (Thermo Fisher, #110808), BECN1 (Thermo Fisher, #137198), UVRAG (Thermo Fisher, #13012), ATG16L1 (Thermo Fisher, 133572), ATG4B (Thermo Fisher, #121660), ATG5 (Thermo Fisher, #6528), ATG7 (Thermo Fisher, #17725), LC3B (Thermo Fisher, #130442) or negative control siRNA (Thermo Fisher, #AM4611) for 3 h. DMEM, high glucose with 20% FBS and 50-µg/ml gentamicin, was added and incubated overnight. The supernatants were replaced with DMEM(+), and the cells were incubated for 48 h. Transfected cells were used for the experiments. RNAi by shRNA was conducted in the same procedure as above. To express shRNA for use in RNAi analysis, targeting oligo for shRNA-mediated knockdown was ligated into pENTR/U6 (Invitrogen, # K494500). The sequences of the oligonucleotides were as follows: ULK1 (Top: 5’-caccgccgcatggacttcgatgagttcgaaaactcatcgaagtccatgcgg-3’, Bottom: 5’-aaaaccgcatggacttcgatgagttttcgaactcatcgaagtccatgcggc-3’, NCBI Reference Sequence: NM_003565.2), FIP200 (Top: 5’-caccgatgcctagaacaactaacgaacgaattcgttagttgttctaggcat-3’, Bottom: 5’-aaaaatgcctagaacaactaacgaattcgttcgttagttgttctaggcatc-3’, NCBI Reference Sequence: NM_001083617.1), and ATG13 (Top: 5’-caccgttggttcaacttagcaatcaacgaattgattgctaagttgaaccaa-3’, Bottom: 5’-aaaattggttcaacttagcaatcaattcgttgattgctaagttgaaccaac-3’, NCBI Reference Sequence: NM_001142673.2).

### Rac1 Activity Assay

The GTPase activity of Rac1 was detected using the Active Rac1 Detection Kit (CST, #8815). Briefly, cells were washed with ice-cold PBS and lysed with 1× lysis/binding/wash buffer that was freshly added with 1-mM PMSF. After centrifugation at 16,000×*g* at 4°C for 15 min, the protein concentration was assayed using the BCA Protein Assay Kit (Wako). Then GST-PAK1-PBD fusion resin beads were added to the supernatant and incubated at 4°C for 1 h. Finally, the resin-bound active Rac1 was eluted with 2× SDS sample buffer containing 200-mM dithiothreitol for Western blotting using Rac1 mAb (CST, #8631, 1:1,000).

### Immunoprecipitation

Cell pellets were washed with Wash buffer [100-mM NaCl, 10-mM Tris–HCl (pH 7.4), 1-mM DTT] and prepared with lysis buffer [100-mM NaCl, 100-mM Tris–HCl (pH 7.4), 0.02% NP-40, 1-mM PMSF]. The lysate was centrifuged at 15,000 rpm at 4°C for 10 min, and the protein concentration of the supernatant was adjusted to 1.0 mg/ml using BCA Protein Assay Kit (Wako). The samples were incubated with antibodies of LC3B (CST, #83506S), Flag (Sigma, #F3165), or normal mouse IgG (SantaCruz, #sc-2025) at 4°C overnight. Then, protein A-Sepharose beads (Cytiva, #17078001) were added and incubated for 90 min at 4°C. The beads were washed with wash buffer, and bound protein were eluted by boiling in SDS sample buffer [100-mM Tris–HCl (pH 6.8), 4% SDS, 10% ß-mercaptmomethanol, 20% glycerol]. After centrifugation, the supernatant was collected and taken out for Western blotting.

### Statistical Analysis

Statistical analysis of all data was conducted using Student’s *t*-test for paired data. Data from 3 independent experiments were evaluated. All tests were one-tailed.

## Data Availability Statement

The original contributions presented in the study are included in the article/**Supplementary Material**. Further inquiries can be directed to the corresponding author.

## Author Contributions

TS, AT, and SF contributed to conception and design of the study. SF, TS, YI, and JK conducted the experiments. SF and TS wrote the first draft of the manuscript. AT, TU, and KM contributed materials and helped to analyze the data. All authors listed have made a substantial, direct, and intellectual contribution to the work and approved it for publication.

## Funding

JSPS KAKENHI (Grant Numbers JP20H01616, JP15K00819, and JP20K11647).

## Conflict of Interest

The authors declare that the research was conducted in the absence of any commercial or financial relationships that could be construed as a potential conflict of interest.

## Publisher’s Note

All claims expressed in this article are solely those of the authors and do not necessarily represent those of their affiliated organizations, or those of the publisher, the editors and the reviewers. Any product that may be evaluated in this article, or claim that may be made by its manufacturer, is not guaranteed or endorsed by the publisher.
