# Supplementary material for: Recruitment of LC3 by Campylobacter jejuni to Bacterial Invasion Site on Host Cells via the Rac1-Mediated Signaling Pathway
Source: Front Cell Infect Microbiol. 2022 Mar 3;12:829682. doi: 10.3389/fcimb.2022.829682 (PMC8927770; doi:10.3389/fcimb.2022.829682)
Supplement: Supplementary file 1 [file DataSheet_1.docx]

Supplementary Material

# Supplementary Figures

**Materials and Method**

**Bacterial strains and culture conditions**

*Salmonella enterica* serovar Typhimurium (*S. typhimurium*) χ3306 strain was cultured in LB medium at 37°C with shaking. For the experiment, bacterial cells were centrifuged at 12,000 rpm for 3 min, washed the pellet with PBS, centrifuged again, and resuspended in PBS to adjust to OD_600_ of 1.0.

**Quantification of RNAi-mediated knockdown efficiency**

The efficiency of siRNA-mediated knockdown was analyzed by Western blotting and detection using antibodies for ULK1 (GeneTex, #GTX115378), Beclin-1 (CST, #3495P), UVRAG (CST, #5320S), ATG16L1 (CST, 8089P), ATG4B (GeneTex, #GTX115678), ATG5 (CST, #12997P), ATG7 (CST, #8558P), LC3 (CST, #12741P), FIP200 (GeneTex, #GTX107387), and ATG13 (GeneTex, #GTX123970). The band intensity of scanned immunoblots was quantified using ImageJ.

**FITC-dextran uptake**

HeLa cells were seeded at a density of 5 × 10^4^ cells/well in 24-well plates and incubated at 37°C for three days for the experiment. The cells were treated with 300-nM cytochalasin D and 7.5-mM MßCD in DMEM (−) for 1 h, and incubated with DMEM (−) containing 100-μg/ml 10 kDa FITC-dextran for 3 h. After that, the cells were washed with ice-cold HEPES buffer (10-mM HEPES, 145-mM NaCl, 10-mM glucose, 5-mM KCl, 1-mM MgCl_2_, 1-mM CaCl_2_) thrice and lysed with RIPA buffer. The cell lysates were centrifuged at 15,000 rpm at 4°C for 10 min, and the supernatant was used for measurement of fluorescence of FITC-dextran by the fluorescence microplate reader (Molecular Devices) at excitation and emission wavelengths of 490 and 520 nm, respectively.

**Invasion assay of *Salmonella***

HeLa cells were seeded at a density of 5 × 10^4^ cells/well in 24-well plates and incubated at 37°C for three days.

The culture medium was replaced with DMEM (−), and the cells were infected with *Salmonella* for 1 h. After the infection, extracellular bacteria were removed and the culture medium replaced 200-µg/mL gentamicin containing DMEM for 2 h. Then, the cells were washed with PBS and lysed with 1% Toriton-X in PBS for 5 min at 37°C. Finally, diluted cell lysates were plated on LB agar plates and incubated for 24 h at 37°C. Intracellular bacterial cell number was determined by counting colony-forming units (CFU), and the bacterial number was normalized with the protein concentration of the individual samples. Protein concentration was measured using BCA Protein Assay Kit (Thermo Fisher).

## Supplementary Figures

**
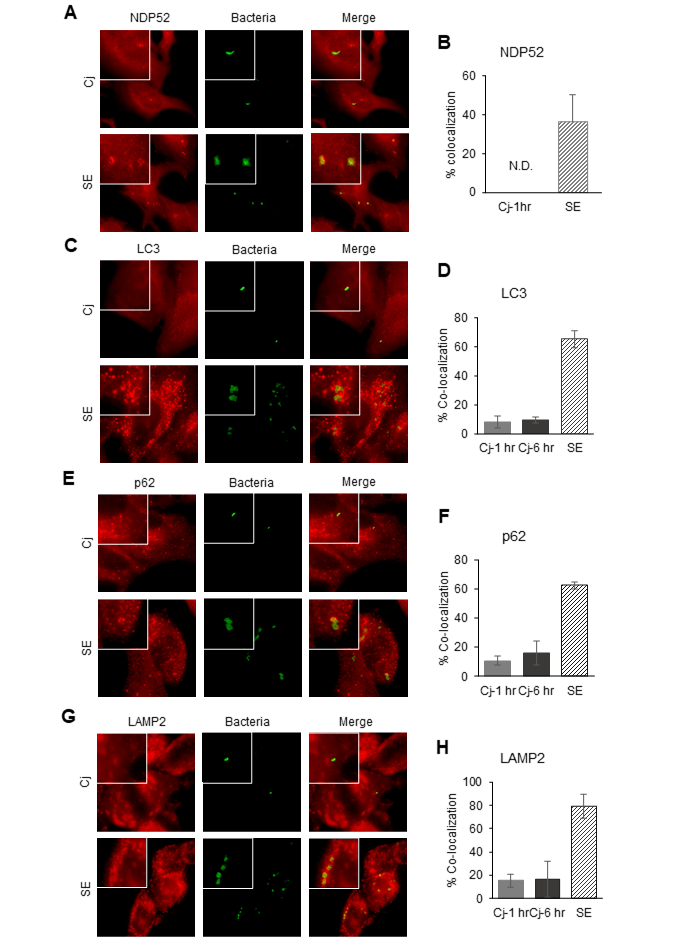
**

**Fig. S1 *C. jejuni* was not colocalized with autophagy organelles.** (A) Representative immunofluorescence images of HeLa cells infected with *S.* Enteritidis (SE) for 1hr or *C. jejuni* (Cj) for 6hr. *S.* Enteritidis and *C. jejuni* was stained with 10 ng/ml 5(6)-Carboxyfluorescein diacetate (CFDA) solution, and infected to HeLa cells. Cells were immunostained by anti-NDP52 antibody, and observed by Keyence BZ-X700 fluorescence microscope. Cj-1 hr: n=22, SE: n=69. (B) Intracellular bacteria which co-localized with NDP52 was quantified. Co-localization rate was determined at 1 hr *p. i.* (C, E, G) Representative immunofluorescence images of HeLa cells infected with *S.* Enteritidis (SE) for 1hr or *C. jejuni* 81-176 strain (Cj) for 6hr. *S.* Enteritidis and *C. jejuni* was stained with 10 ng/ml CFDA solution, and infected to HeLa cells. Cells were immunostained by anti-LC3(C), p62 (E), and LAMP2 (G) antibody, and observed by Keyence BZ-X700 fluorescence microscope. (D, F, H) Intracellular bacteria which co-localized with LC3 (Cj-1 hr: n=54, Cj-6 hr: n=43, SE: n=277) (B), p62 (Cj-1 hr: n=55, Cj-6 hr: n=56, SE: n=205) (D), and LAMP2 (Cj-1 hr: n=48, Cj-6 hr: n=44, SE: n=152) (F) were quantified. Co-localization rate was determined at 1 hr *p. i.* (Cj-1 hr) and 6 hr *p. i.* (Cj-6 hr) in *C. jejuni* 81-176 strain infection and 1 hr *p. i.* (SE) in *S.* Enteritidis infection. All data are expressed as means ± standard deviations from t 3 independent experiments. Differences were evaluated with Two-tailed Student's t-test. **p* < 0.05, ** *p* < 0.001.


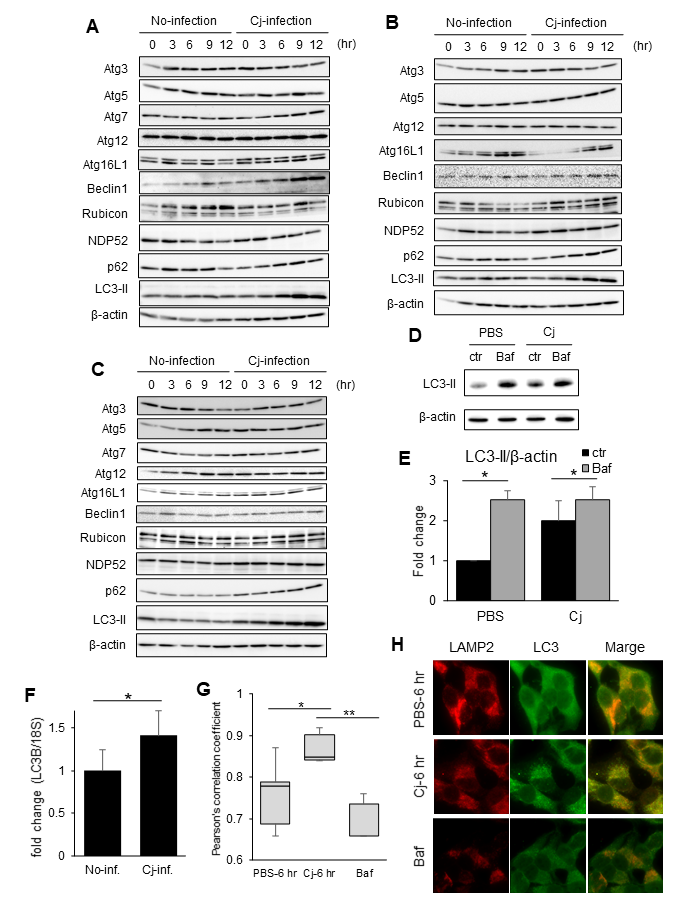


**Fig. S2 Autophagy signaling in *C. jejuni* infection.** (A, B, C) HeLa cells were infected with *C. jejuni* 11168 strain (A)*,* 81-176 strain (B), 81116 strain (C), respectively, and autophagy-associated proteins were detected. (D) HeLa cells were treated by 200 nM Bafilomycin A1 for 6 hr with or without *C. jejuni* infection, and LC3-II level was confirmed by Western blotting. (E) LC3-II level was quantified and normalized by protein level of β-actin. (F) Relative fold change of LC3B mRNA level was determined by quantitative reverse transcription PCR (qRT-PCR) analysis for no-infected HeLa cells (No-inf.) or *C. jejuni*-infected cells (Cj-inf.) at 6 hr *p. i..* All data are expressed as means ± standard deviations from t 3 independent experiments. (G) HeLa cells were infected with *C. jejuni* for 6 hr or treated with 200 nM Bafilomycin A1 (Baf) for 3 hr. Colocalization rate between LC3 and LAMP2 was determined by Pearson’s correlation coefficient using ImageJ. n=6. (H) The microscope data indicated representative immunofluorescence images of (G). HeLa cells were stained by anti-LC3 and anti-LAMP2, and observed by Keyence BZ-X700 fluorescence microscope. Differences were evaluated with Two-tailed Student's t-test. **p* < 0.05, ** *p* < 0.001.


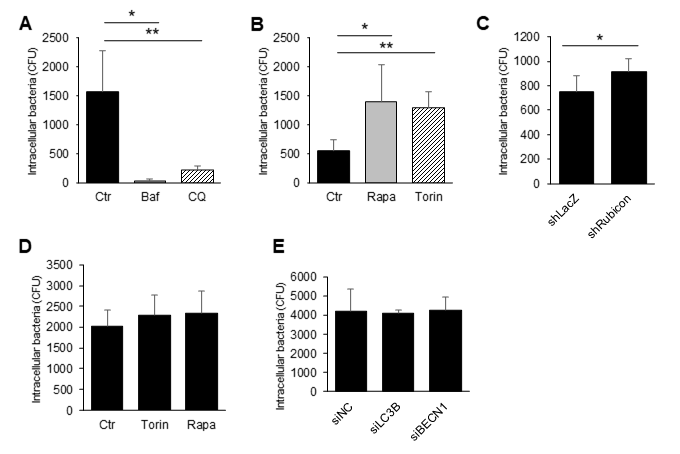


**Fig. S3 Effect of autophagy inhibition or induction in *C. jejuni* invasion in Caco-2 cells or *S*. Enteritidis invasion in HeLa cells.** (A) Caco-2 cells were treated with 200 nM Bafilomycin A1 (Baf) or 50 μM Chloroquine (CQ) for 3 hr, and the number of invaded *C. jejuni* was estimated by gentamicin protection assay at 1 hr *p. i.* (B) The number of intracellular *C. jejuni* in Caco-2 cells, which treated by 1 μM Torin 1 (Torin) or 200 nM Rapamycin (Rapa) for 3 hr, were measured by gentamicin protection assay at 1 hr *p. i.* (C) HeLa cells were transfected by shRNA-control (shLacZ) or shRNA for Rubicon (shRubico). The number of intracellular *C. jejuni* was estimated by gentamicin protection assay at 1 hr *p. i.* (D) HeLa cells were treated with 1 μM Torin 1 (Torin) or 200 nM Rapamycin (Rapa) for 3 hr, and the number of intracellular *S.* Enteritidis was estimated by gentamicin protection assay at 1 hr *p. i.* (E) HeLa cells were transfected by siNegative control (siNC), siLC3B, and siBECN1. The number of intracellular *S.* Enteritidis was estimated by gentamicin protection assay at 1 hr *p. i.*

*
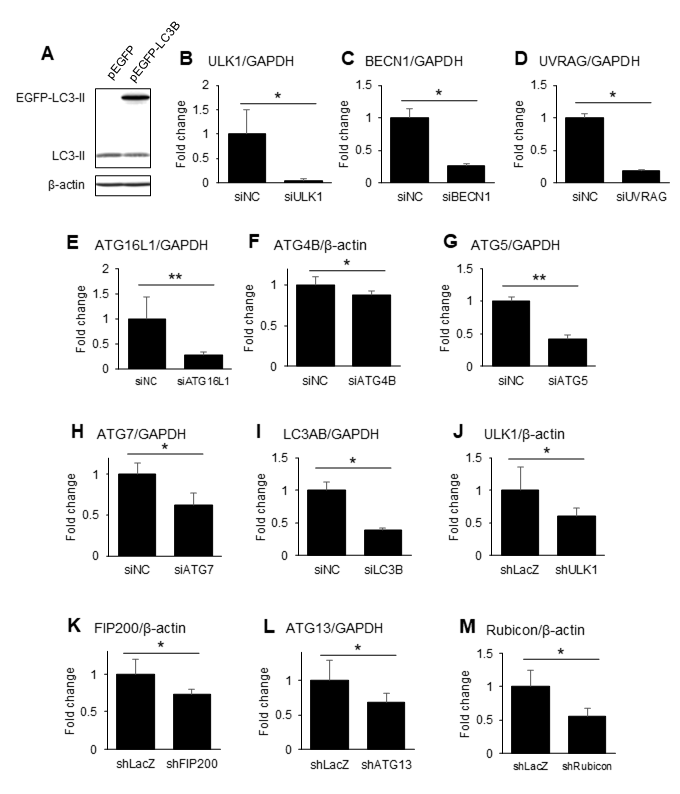
*

**Fig. S4 The efficiency of RNAi knock down in HeLa cells.** (A) The expression of LC3 level was confirmed in pEGFP or pEGFP-LC3B stably expressed HeLa cells by Western blotting analysis. (B-M) HeLa cells were transfected by Negative control siRNA (siNC) or siRNA for autophagy-related genes, and the efficiency of knockdown was confirmed by quantification of band intensity of Western blotting analysis. (J-M) HeLa cells were transfected by shLacZ or shRNA for autophagy-related genes. The efficiency of knock down was confirmed by Western blotting and band intensity was quantified. All data are expressed as means ± standard deviations (n=4). Differences were evaluated with Two-tailed Student's t-test. **p* < 0.05, ** *p* < 0.001.


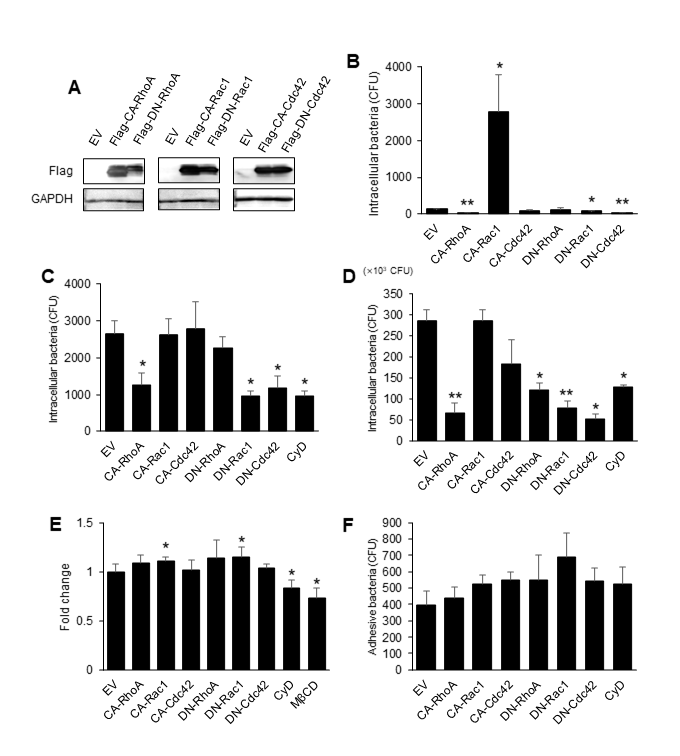


**Fig. S5 Effect of actin dynamics regulated by small GTPases to *C. jejuni* infection.** (A) The expression level of constitutive active form (CA) and dominant negative form (DN) of Flag-tagged RhoA, Rac1, and Cdc42 were confirmed by Western blotting. (B) CA and DN of RhoA, Rac1, and Cdc42 was transfected in HeLa cells. The number of intracellular *C. jejuni* 81116 strain was estimated by gentamicin protection assay at 1 hr *p. i.* (C) HeLa cells were expressed CA and DN of RhoA, Rac1, and Cdc42, or treated with 300 nM Cytochalasin D (CyD) for 1 hr. The number of intracellular *S.* Enteritidis was estimated by gentamicin protection assay at 1 hr *p. i.* (D) CA and DN of RhoA, Rac1, and Cdc42 expressed HeLa cells were infected with *S.* Typhimurium for 1 hr. The number of intracellular bacteria at 1 hr *p. i.* was measured by gentamicin protection assay. (E) HeLa cells were transfected as shown or treated with 300 nM CyD or 7.5 mM MβCD for 1 hr. The cells were treated with FITC-dextran at 200 μg/mL, and uptake of FITC-dextran was measured by fluorescence intensity of cell lysates. (F) CA and DN of RhoA, Rac1, and Cdc42 was transfected in HeLa cells. Cells were infected with *C. jejuni*, and bacterial adherence to cells was measured. All data are expressed as means ± standard deviations from 3 independent experiments. Differences were evaluated with Two-tailed Student's t-test. **p* < 0.05, ** *p* < 0.001.


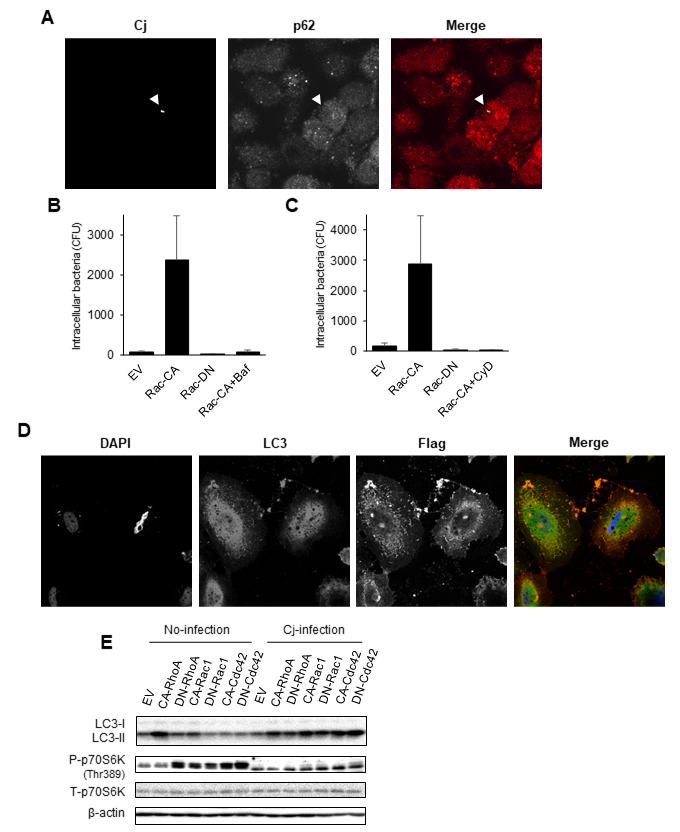


**Fig. S6 Signaling induction in CA-Rac1 expressed HeLa cells.** (A) CA-Rac1 expressed HeLa cells were infected with CFDA-stained *C. jejuni* (green) for 20 min. Cells were immunostained by anti-p62 antibody (red).　(B, C) EV, CA-Rac1, DN-Rac1 was transfected to HeLa cells. CA-Rac1 expressed cells were treated with 200 nM Bafilomycin A1 (Baf) for 3 hr or 300 nM Cytochalasin D (CyD) for 1 hr, and invasion rate of *C. jejuni* was estimated by gentamicin protection assay at 1 hr *p. i.* (D) HeLa cells were transfected CA-Rac1 (red), and immunostained anti-LC3 antibody (green) and DAIP (blue). (E) CA and DN of RhoA, Rac1, and Cdc42 was transfected in HeLa cells. LC3-II protein level and phosphorylation level of p70S6K was analyzed by Western blotting at 12 hr *p.i..* All data are expressed as means ± standard deviations from 3 independent experiments. Differences were evaluated with Two-tailed Student's t-test. **p* < 0.05, ** *p* < 0.001.
